# Supplementary material for: Initiation of human cytomegalovirus secondary envelopment requires the gM/gN glycoprotein complex and involves palmitoylation
Source: J Virol. 2025 Dec 8;100(1):e01567-25. doi: 10.1128/jvi.01567-25 (PMC12817960; doi:10.1128/jvi.01567-25)
Supplement: Supplemental figures — Figures S1 to S4. [file jvi.01567-25-s0001.pdf]

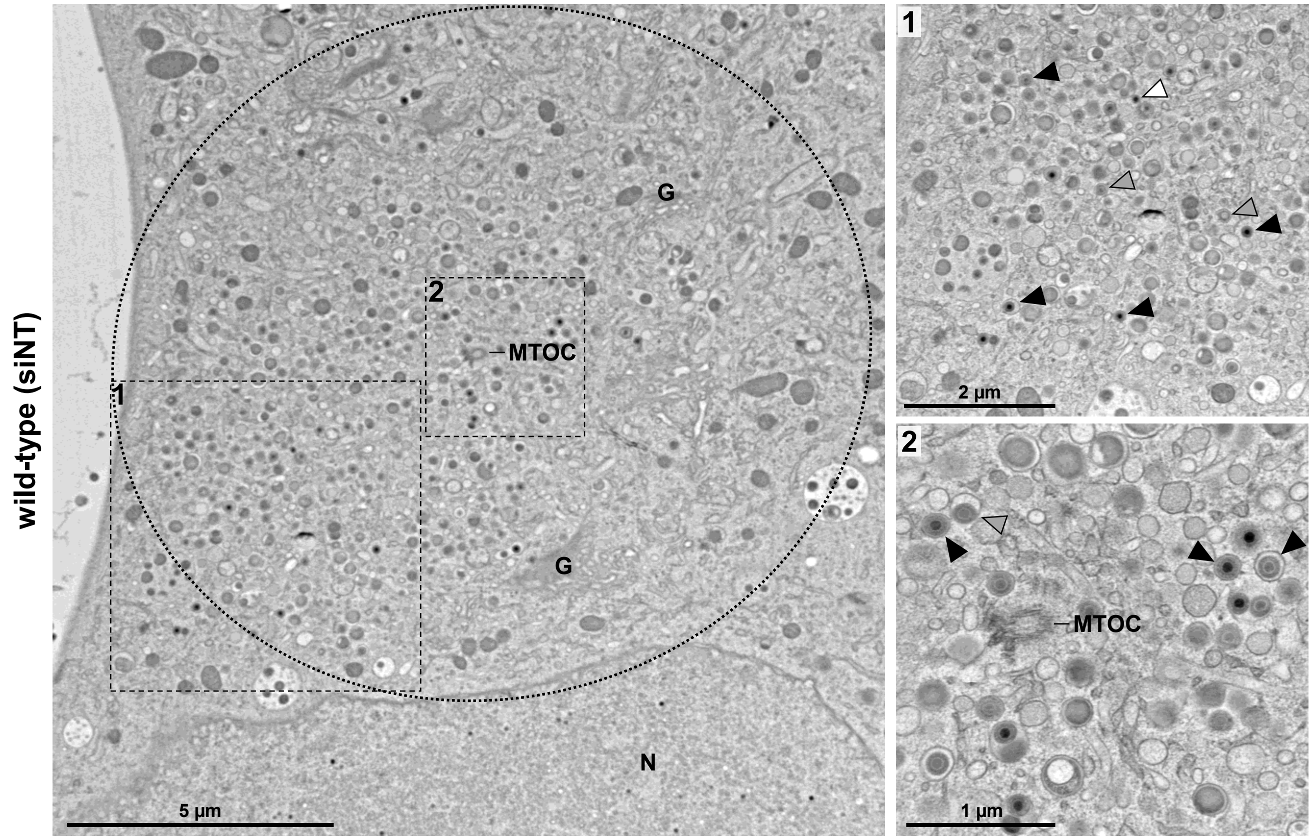

Fig. S1. The electron microscope image of a representative cVAC from wild-type-infected HFFs, 120 h post-infection, transfected with non-targeted siRNA (siNT), showed no ultrastructural differences compared to the cVAC from non-transfected, wild-type-infected control cells. The cVAC is indicated with a dashed circle. Dashed boxes show higher magnifications of selected areas. Capsids are labeled according to their envelopment stage: free capsids (white arrowheads), budding capsids (gray arrowheads), and enveloped capsids (black arrowheads). N, nucleus; G, Golgi; MTOC, microtubule-organizing center.

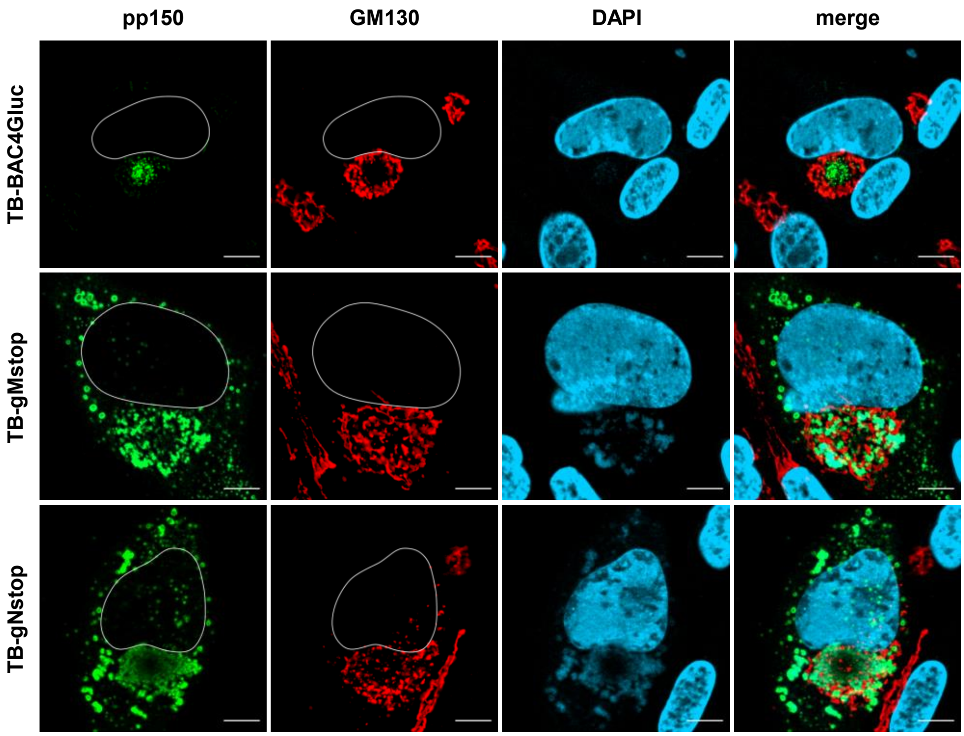

Fig. S2. Localization of capsid-associated tegument protein pp150 (green) within the cVAC (red), detected by staining for the cis-Golgi protein GM130 in TB-BAC4Gluc, TB-gNstop, and TB-gMstop infected fibroblasts at 120 h post-infection. Cell nuclei were stained with DAPI (blue) and outlined in white. Scale bars, 10 µm.

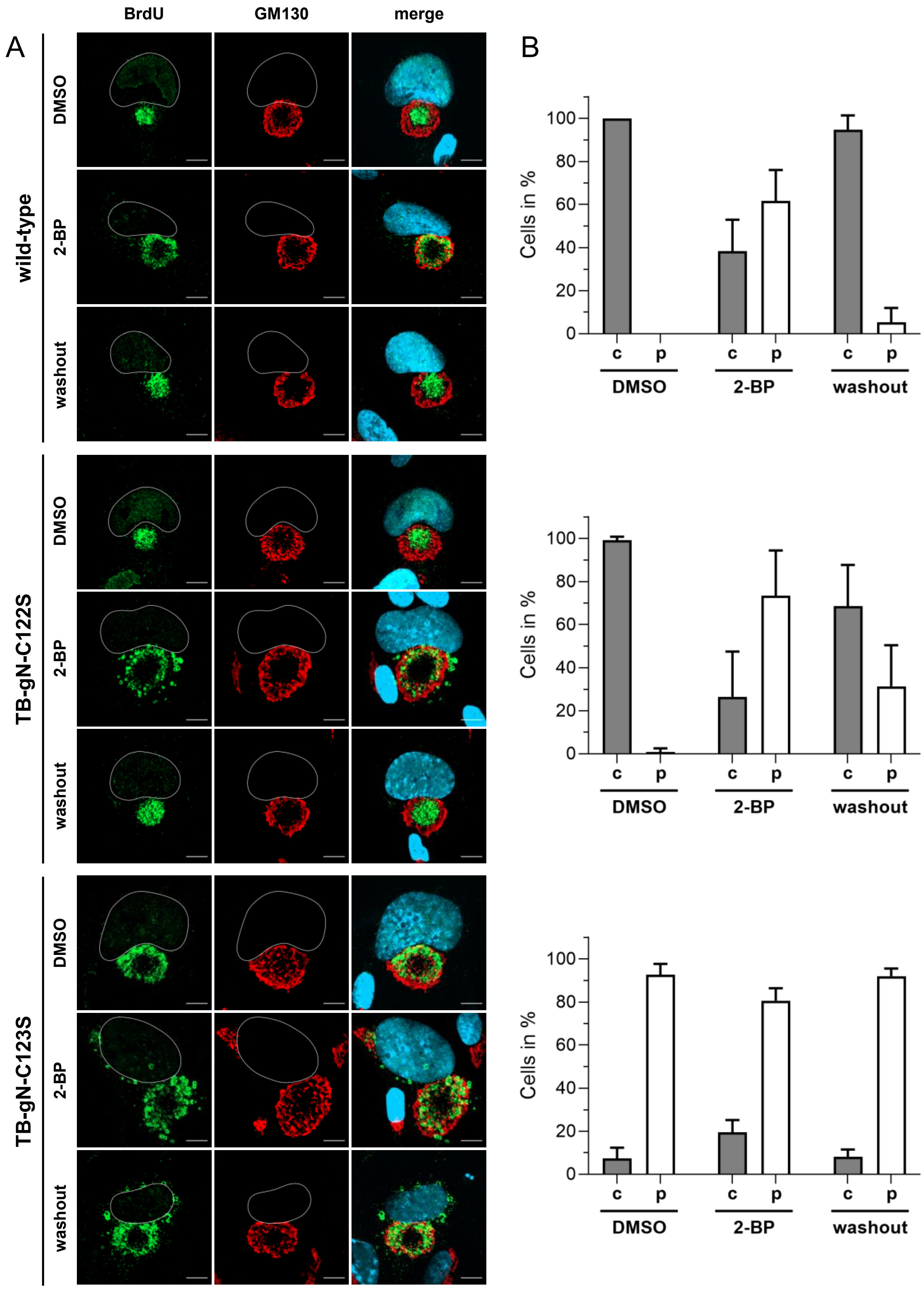

Fig. S3. The central distribution of nucleocapsids at the cVAC is restored after the palmitoylation inhibitor 2-BP is washed out. (A) Indirect immunofluorescence of HFFs infected with wild-type, TB-gN-C122S, and TB-gN-C123S at 120 h post-infection. The cells were treated with 2-BP, DMSO, or 2-BP until 96 h post-infection, after which 2-BP was removed (washout). Nucleocapsids were detected by pulse labeling viral genomes with BrdU (green) at 96 h post-infection. The cVAC was stained with the cis-Golgi marker GM130 (red). Cell nuclei were stained with DAPI (blue) and outlined in white. Scale bars, 10 µm. (B) Quantification of BrdU signal distribution within the cVAC under DMSO, 2-BP, and washout conditions in wild-type, TB-gN-C122S, and TB-gN-C123S infected fibroblasts. Shown are the mean percentage and standard deviation of cells with a central BrdU distribution (gray) and a peripheral BrdU distribution (white). c, center; p, periphery.

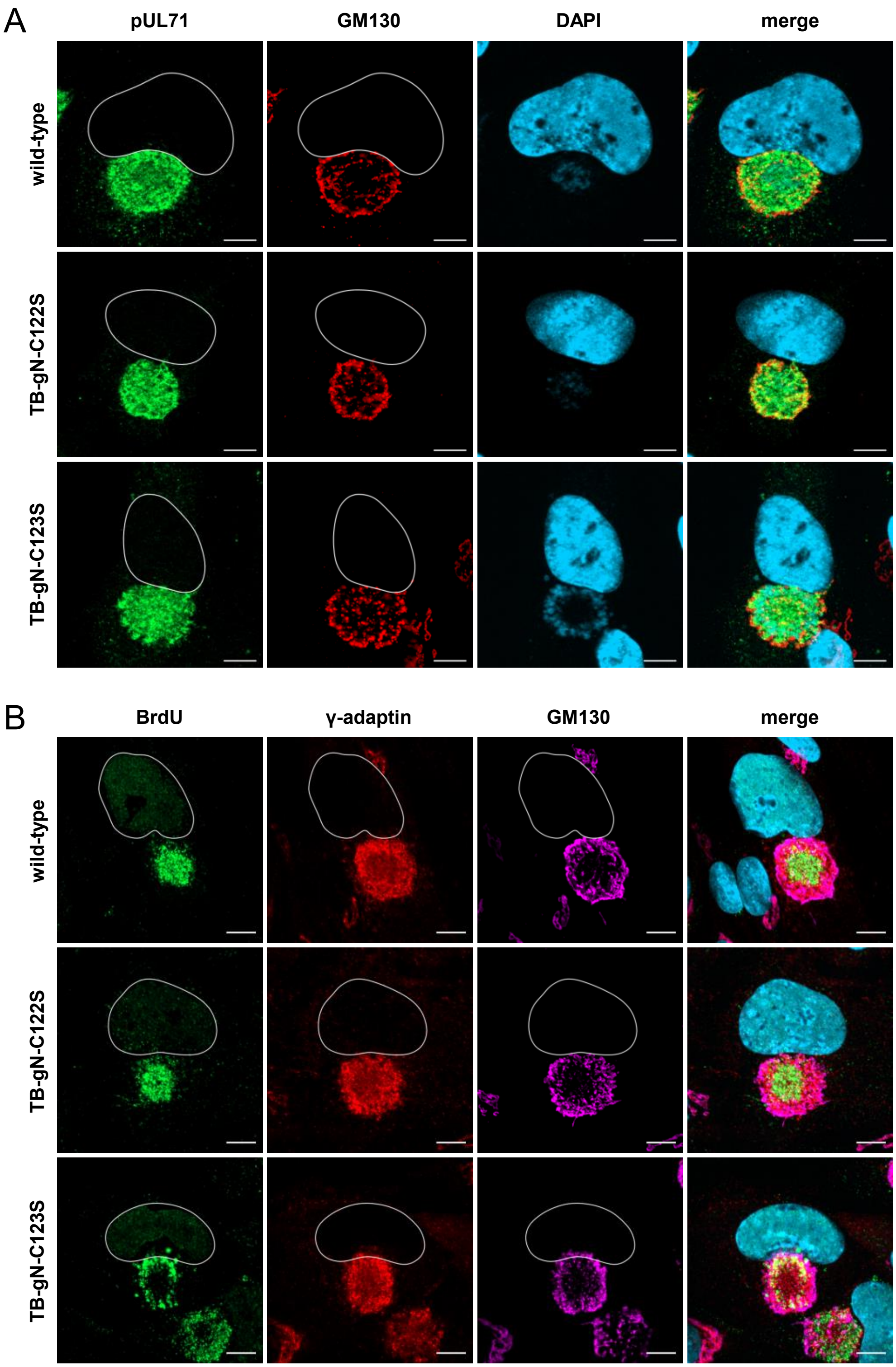

Fig. S4. Localization study of viral and cellular components at the cVAC in wild-type, TB-gN-C122S, and TB-gN-C123S infected fibroblasts at 120 h post-infection. (A) Distribution of tegument protein pUL71 (green) within the cVAC (red), detected by staining for the cis-Golgi protein GM130. (B) Localization of trans-Golgi cellular marker γ-adaptin (red) within the cVAC (magenta). Nucleocapsids were detected through pulse labeling with BrdU (green). Cell nuclei were stained with DAPI (blue) and outlined in white. Scale bars, 10 µm.
